# A Cantilever Beam-Based Triboelectric Nanogenerator as a Drill Pipe Transverse Vibration Energy Harvester Powering Intelligent Exploitation System

**DOI:** 10.3390/s22114287

**Published:** 2022-06-04

**Authors:** Zhenhui Lian, Qunyi Wang, Chuanqing Zhu, Cong Zhao, Qiang Zhao, Yan Wang, Zhiyuan Hu, Ruijiang Xu, Yukai Lin, Tianyu Chen, Xiangyu Liu, Xiaoyan Xu, Ling Liu, Xiu Xiao, Minyi Xu

**Affiliations:** Dalian Key Lab of Marine Micro/Nano Energy and Self-Powered Systems, Marine Engineering College, Dalian Maritime University, Dalian 116026, China; zhlian_bkpp@dlmu.edu.cn (Z.L.); 1120211168@dlmu.edu.cn (Q.W.); zcq@dlmu.edu.cn (C.Z.); zhaocong@dlmu.edu.cn (C.Z.); zhao1989@bcnu.edu.cn (Q.Z.); wangyanme@dlmu.edu.cn (Y.W.); zhiyuanhu@dlmu.edu.cn (Z.H.); xuruijiang@dlmu.edu.cn (R.X.); linyukai@dlmu.edu.cn (Y.L.); cty@dlmu.edu.cn (T.C.); simonlxy@dlmu.edu.cn (X.L.); yz1769135517@dlmu.edu.cn (X.X.); pinky@dlmu.edu.cn (L.L.); xuminyi@dlmu.edu.cn (M.X.)

**Keywords:** triboelectric nanogenerator, cantilever beam, transverse vibration, drill pipe, measurement while drilling

## Abstract

Measurement While Drilling (MWD) is the most commonly used real-time information acquisition technique in offshore intelligent drilling, its power supply has always been a concern. Triboelectric nanogenerators have been shown to harvest low-frequency vibrational energy in the environment and convert it into electricity to power small sensors and electrical devices. This work proposed a cantilever-beam-based triboelectric nanogenerator (CB-TENG) for transverse vibration energy harvesting of a drill pipe. The CB-TENG consists of two vibrators composed of spring steel with PTFE attached and Al electrodes. The structurally optimized CB-TENG can output a peak power of 2.56 mW under the vibration condition of *f* = 3.0 Hz and *A* = 50 mm, and the electrical output can be further enhanced with the increased vibration parameters. An array-type vibration energy harvester integrated with eight CB-TENGs is designed to fully adapt to the interior of the drill pipe and improve output performance. The device can realize omnidirectional vibration energy harvesting in the two-dimensional plane with good robustness. Under the typical vibration condition, the short-circuit current and the peak power can reach 49.85 μA and 30.95 mW, respectively. Finally, a series of demonstration experiments have been carried out, indicating the application prospects of the device.

## 1. Introduction

The ocean is abundant in oil and gas resources, which occupies 43.7% of global reserves [1]. However, it is very technically challenging to exploit oil and gas resources because of the changing marine environment. Intelligent mining technology, which relies on the Measurement While Drilling (MWD) system to obtain real-time underground conditions and adjusts the drilling plan accordingly, is suitable for the exploitation of oil and gas resources [2]. Nevertheless, the power supply of MWD still faces some challenges. Currently, there are mainly three power supply modes for MWD, namely, the lithium battery, downhole turbine generator, and cable laying, all of which have merits and demerits. To be specific, it is convenient to use a lithium battery, but the drilling cycle is limited by the battery capacity, and the disposal of batteries can cause severe environmental pollution [3]. The use of a downhole turbine generator prolongs the drilling cycle, but it has strict requirements for the physical property parameters of the slurry [4]. Further, laying cables makes it harder for the process of drilling and the design of the drill pipe [5]. Hence, it is necessary to explore a new method of power supply, which can serve as a supplement or be combined with the existing power supply methods, so as to promote the development of intelligent drilling.

With the development of intelligence, self-powered technology has received extensive attention from researchers. Energy harvesting from the ambient environment is an effective method for building up self-powered systems. During the drilling process, the drill pipe will generate strong vibration and cause significant energy loss, which increases the power burden of MWD. Therefore, it is promising to utilize energy-harvesting technology to scavenge the vibration energy of the drill pipe to power small electrical devices and distributed sensors in the drill pipe. Numerous vibration energy harvesters have been reported for capturing various forms of vibration energy, such as electromagnetic generators [6,7,8,9], piezoelectric generators [10,11,12], and triboelectric nanogenerators [13,14]. However, due to the high resonant frequency and narrow bandwidth, piezoelectric vibration energy harvesters are not suitable for scavenging low-frequency vibration energy [15,16,17]. Moreover, the large-scale and complex structures of electromagnetic vibration energy harvesters make them unsuitable for applications where small space and maintenance-free operation are needed.

Recently, triboelectric nanogenerator (TENG) technology [18,19,20,21,22,23,24,25] based on the combination of contact electrification and electrostatic induction has been invented. Researchers have reported that it has numerous advantages, including high energy conversion efficiency at a low frequency, small size, low cost, multiple working modes, material diversity, and multi-field applications [26]. Until now, several vibration energy triboelectric nanogenerator (VE-TENG) harvesters have been proposed [27,28,29,30,31,32,33,34]. According to the structural characteristics, the VE-TENG can be roughly divided into three types, which are the spring-assisted structure, inertia ball structure, and multi-layer composite structure. For instance, Li et al. [31] proposed a contact-mode TENG, which consists of a tribo-pair, two electrodes, two acrylic plates, several springs, and a waterproof package. The output power of TENG reached the maximum value of 14.0 µW with a power density of 5.56 mW/m^2^ at the frequency of 8 Hz and amplitude of 4 mm. Xu et al. [29] designed an S-TENG integrated with a spring by fabricating a helical structure along the spring. Under vertical resonance vibration (16 Hz) and horizontal resonance vibration (8.5 Hz), the average power density of the presented S-TENG was found to be 240 and 45 mW/m^2^, respectively. As for the inertia ball structure, Xiao et al. [32] designed a honeycomb structure TENG in which each groove was filled with a polytetrafluoroethylene ball. The output power density was 50 W/m^3^ under the frequency of 25 Hz and the amplitude of 2 mm. Wang et al. [13] developed a stackable triboelectric nanogenerator(S-TENG), with each layer made into multiple channels carrying polytetrafluoroethylene balls in between Aluminum electrodes. The peak power density of the S-TENG reached 49 W/m^3^ under the frequency of 2 Hz and the amplitude of 150 mm. Considering the motion characteristic of the cantilever beam, Yang et al. [30] demonstrated a triple-cantilever-based triboelectric nanogenerator for harvesting ambient vibration energy. Three metal plates of beryllium-copper foils served as the three cantilevers. The bottom surface of the top cantilever and the top surface of the bottom cantilever were coated with PDMS films. The surfaces of the middle cantilever were covered in ZnO nanowire arrays, on the top of which a layer of Cu was deposited. A mass was also attached to its end for the effectiveness of vibration. The open-circuit voltage and rectified short-circuit current reached approximately 101 V and 55.7 μA, respectively, with a peak power density of 252.3 mW/m^2^ under a vibration frequency of 3.7 Hz. Liu et al. [35] designed a Newton’s Cradle motion-like triboelectric nanogenerator (NC-TENG) based on three elastic plates, with one acrylic board in the center and two identical steel plates on both sides. The output of NC-TENG was 470% higher than that of the common contact-separation TENG and 130% higher than that of a similar-structure TENG without using elasticity. Under the frequency of 2.5 Hz and the amplitude of 8 mm, the maximum short-circuit current, open-circuit voltage, and output power were 114 μA, 428 V, and 4.32 W/m^2^, respectively. The conditions and performance of the TENG-based vibration harvester mentioned are summarized in Table 1.

**Figure 1:**
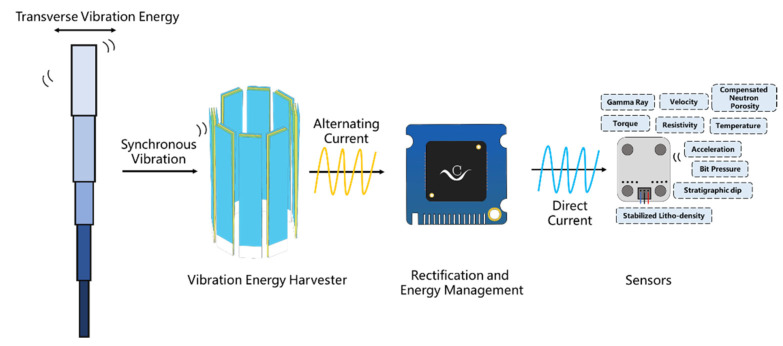
The concept diagram of this work.

From the above studies, it can be seen that the resonance frequency of the fundamental flexural modes of a cantilever is much lower than that of other vibration structures, and it can convert low-frequency vibration energy into electrical output effectively [36]. Considering the limited space of the interior drill pipe and the low frequency, as well as the low amplitude, of the drill pipe vibration, this work proposes a cantilever-beam-based triboelectric nanogenerator (CB-TENG), which contains double cantilever beams. To further enhance the electrical output, the CB-TENG is integrated to form an array-type vibration energy-harvesting device. Results show that the distinguished output performance can power small electrical devices and disturbed sensors in the drill pipe, which is expected to promote offshore intelligent exploitation. The concept diagram is shown in Figure 1.

## 2. Materials and Methods

**Figure 2:**
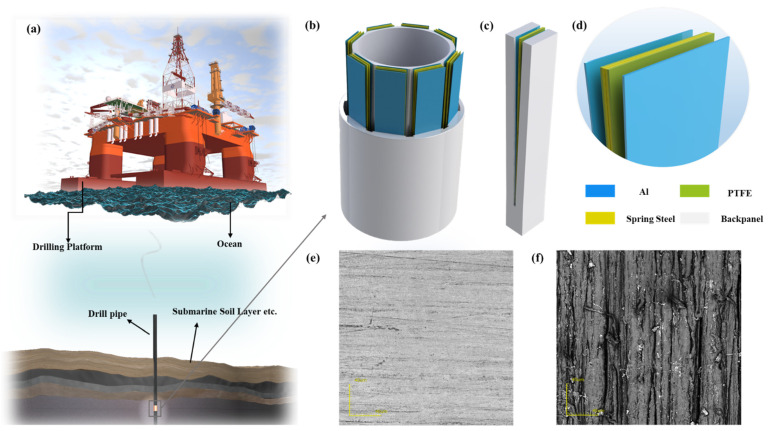
Application scenario and structure of CB-TENG device (**a**) Application of CB-TENG in vibration energy collection of drill pipe for offshore oil extraction; (**b**) the structure of array-type CB-TENG; (**c**) the composition of CB-TENG; (**d**) the partial enlargement of the tip of CB-TENG; (**e**) SEM image of PTFE surface (Not sanded); (**f**) SEM image of sanded PTFE surface.

The application scenario of CB-TENG for energy harvesting of the transverse vibration of subsea drill pipes is shown in Figure 2a. During subsea drilling, the drill pipe will produce periodic vibration under the strike of rocks. In general, the vibration frequency and amplitude of the transverse vibration range from 0 to 3.0 Hz and 0 to 50 mm, respectively. As exhibited in Figure 2b, 8 CB-TENGs are highly integrated along the circumference to form an array-type vibration energy-harvesting device so as to make full use of the internal space of the drill pipe and improve the electrical output. The as-proposed vibration energy-collection device harvests vibration energy by synchronizing transverse vibration with a drill pipe. The circumferential array can realize the harvesting of multi-directional vibration energy on the two-dimensional plane. Besides, such a hermetic design can reduce the influence of humidity on the performance of triboelectric nanogenerators in the marine environment.

Figure 2c presents the structure of CB-TENG. The backplanes and card slots of CB-TENG are manufactured by a 3D printer using polylactic acid (PLA). The two backplanes are arranged obliquely and cross-connected at the card slot. An aluminum membrane with a thickness of 0.08 mm is attached to the backplane as the metal electrode. Two vibrators are clamped in the card slot to form the cantilever beam structure, and the vibrators are composed of PTFE (a length of 200 mm, width of 30 mm, and thickness of 0.08 mm) and spring steel sheets (a length of 200 mm, width of 30 mm, and thickness of 0.12 mm). Notably, the back-to-back stacked vibrators can effectively increase the contact area of triboelectric layers, thereby enhancing electrical output. The main structural parameters of the CB-TENG are determined by the size of the conventional subsea drill pipe. Specifically, the bottom and top spacings are 3 mm and 10 mm, respectively, and the thickness of the backplane is 5 mm while the width is 33 mm. The locally enlarged details of the CB-TENG are shown in Figure 2d, which clearly reveals the arrangement of the triboelectric pairs. Moreover, it has been demonstrated that nanostructures can improve the surface roughness of the triboelectric layers and increase the effective contact area between them [37]. Therefore, the surface of PTFE is sanded with 10,000 mesh sandpaper in this work. The original and sanded PTFE surfaces are shown in Figure 2e,f, respectively. It can be clearly seen that the sanded PTFE surface has surface nanostructures and is much rougher.

During the experiment, the CB-TENG is driven by a LinMot E1100-RS liner motor to simulate the transverse vibration of the drill pipe. The maximum frequency is 5.0 Hz. A Keithley 6514 electrometer is used to measure the short-circuit current (*I_sc_*), open-circuit voltage (*V_oc_*), and transferred charge (*Q_sc_*).

## 3. Results and Discussions

### 3.1. Performance of CB-TENG

**Figure 3:**
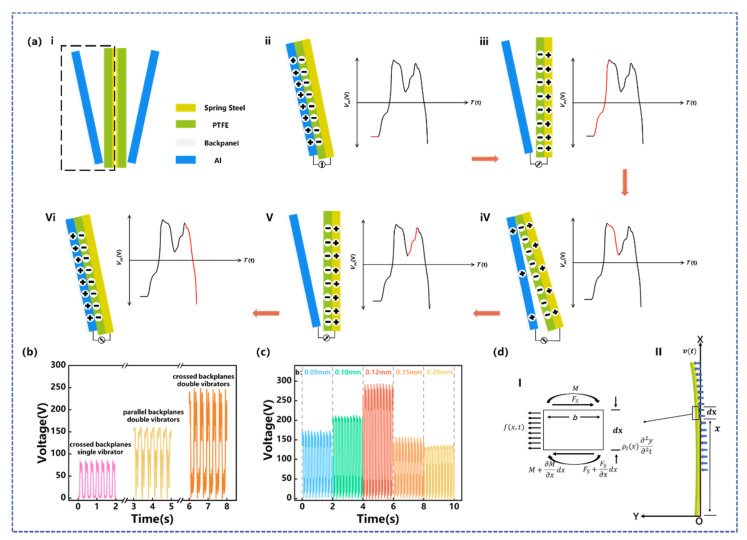
(**a**) Working principle of CB-TENG; (**b**) diagram of CB-TENG voltage output under different backplane structures and different number of vibrators; (**c**) diagram of CB-TENG voltage output at *f* = 3.0 Hz, *A* = 50 mm with different thickness of spring steel; (**dI**) force analysis diagram of unit length vibrator section; (**dII**) schematic diagram of single vibrator overall force.

The CB-TENG works based on the conductor-to-dielectric contact-separation mode of the Al electrode with the dielectric PTFE. During the drilling process, the transverse vibration excites the free end of the cantilever beam to vibrate. The specific vibration mode of the cantilever beam can be found in Video S1 in Supplementary Materials. The PTFE contacts/separates from the Al electrode twice in one cycle, thus generating electrical output. Figure 3a shows the working principle of CB-TENG. Due to the symmetry of the two vibrators, only one vibrator is used here to illustrate the working principle of CB-TENG. As depicted in Figure 3ai, the PTFE is not in contact with the Al electrode in the initial state, and a certain gap is maintained in the middle. When CB-TENG is forced to vibrate by the liner motor, the spring steel with attached PTFE comes into contact with the Al electrode, resulting in equal amounts of positive and negative charges generated on the PTFE surface and the aluminum electrode, as shown in Figure 3aii. Subsequently, the spring steel rebounds to a certain position and separates from the Al electrode to generate a potential voltage, as displayed in Figure 3aiii. Before the vibrator returns to its initial state, the PTFE makes secondary contact with the Al electrode under the influence of the inertia of the spring steel. It can be seen from Figure 3aiv that the charge flows back to the Al electrode at this stage. After that, the PTFE is separated from the Al electrode again, and the induced charges on the Al electrode are completely neutralized by the induced electrons (Figure 3av). Finally, the external load drives the spring steel to contact the Al electrode again, creating a reverse current, as shown in Figure 3avi. The corresponding voltage pulse of CB-TENG is also exhibited in Figure 3. Therefore, the transformation from mechanical energy to electrical output is accomplished through the alternating movement of the spring steel cantilever beam. Furthermore, a finite element analysis is performed using COMSOL Multiphysics to illustrate the working principle of CB-TENG. Figure S1 shows the simulated potential distribution between the PTFE film and the Al electrode in different positions, which is consistent with the above analysis.

In fact, the geometry of CB-TENG, including the structure of the backplanes and the number and thickness of the vibrators, will directly affect its electrical output. In this study, two backplane structures are designed, namely parallel and cross. Among them, the upper and bottom spacings of the parallel backplanes are both 10 mm, while the upper and bottom spacing of the crossed backplanes is 10 mm and 3.0 mm, respectively. In addition, to further enhance electrical output, two vibrators are set in the CB-TENG. Different from the single vibrator, only the side of the spring steel facing the Al electrode is covered with PTFE in the double vibrator structure.

The effects of the backplane structure and the number of vibrators on the output performance of CB-TENG are shown in Figure 3b. At the vibration condition of *f* = 3.0 Hz and *A* = 50 mm, the output voltage of the CB-TENG with the crossed-backplanes and double-vibrator structure reaches approximately 250 V, which is 2.9 times that of the crossed-backplanes and one-vibrator structure and is 1.55 times that of the parallel-backplanes and double-vibrator structure. This is because the deflection of the cantilever beam is a curve that increases gradually with the constraint distance, and the displacement at the top is the largest. In the parallel-backplane structure, the bottom of the vibrator cannot be in contact with the Al electrode, resulting in a small contact area between them. In contrast, for the crossed-backplane structure, the vibrator can make sufficient contact with the Al electrode, thereby increasing the electrical output of CB-TENG. Furthermore, according to *V_oc_ =* *σx(t)/ε*_0_ (where *ε*_0_ is the dielectric constant of vacuum), the charge density *σ* induced by the double vibrators is much larger than that generated by the single vibrator, so the *V_oc_* of the CB-TENG with double vibrators is much higher. Therefore, the crossed backplanes and double vibrators are identified as the basic structure of CB-TENG.

For the vibrator of a certain material, the stiffness is determined by its thickness, which further affects the vibration characteristics of the cantilever beam. Therefore, the thickness of the spring steel is a crucial factor that determines the electrical output of CB-TENG. The deflection displacement of the rigid vibrator can be calculated by the following formula:

(1) $$y=-\frac{mx^{2}}{2K}$$

(2) K=EI

(3) $$I=\frac{ab^{3}}{12}$$

Here, *y* is the deflection displacement of the cantilever beam, *x* is the distance from the origin to the constraint segment, *K* is the stiffness, *E* is elastic modulus, *I* is the moment of inertia of the section, *a* is the width, and *b* is the thickness. It can be seen from the above equation that the stiffness *E* of the spring steel is proportional to the cube of its thickness *b* and the deflection displacement *y* decreases rapidly with the increase in thickness.

Since the very thin spring steel is prone to bending deformation, five spring steels with thicknesses of 0.09 mm, 0.10 mm, 0.12 mm, 0.15 mm, and 0.20 mm were selected for sensitivity analysis in the experiment. Under the vibration frequency of 1.0 Hz, the spring steel with a thickness of 0.09~0.12 mm is deformed by shear force and comes into contact with the Al electrode to generate charge transfer. A clear open-circuit voltage peak can be observed in Supplementary File S2, and it weakens with the increasing thickness. However, under the thickness conditions of 0.15 mm and 0.20 mm, the vibration amplitude is not large enough to make a contact condition, so there is no peak open-circuit voltage. The weak electrical signals at this time are all generated by self-flutter.

It is worth noting that with the increase in the vibration frequency, the external load will cause the forced vibration of the cantilever beam, which, in turn, changes the electrical output characteristics of CB-TENG. The vibrator has a high aspect ratio and can be simplified as a Euler–Bernoulli beam. Assuming that the central inertia axis of each section of the beam is in the same plane and the external load acts in the plane, the beam will be in a free-vibration or forced-vibration state due to different stiffness, and the deformation of the beam is mainly bending deformation. Take the micro-segment of the section at distance *x* from the origin as an example. As shown in Figure 3d, the micro-segment is subjected to shear force *F_S_*, bending moment *M*, inertial force *ρ_l_*(*x*)∙*∂^2^y/∂t* and external force *f*(*x,t*) generated by vibration. According to D ‘Alembert’s principle, the transverse displacement of the section at time *t* can be written as:

(4) $$\rho_{l}\left(x\right)\frac{\partial^{2}y}{\partial t^{2}}dx=F_{S}-\left(F_{S}+\frac{\partial F_{S}}{\partial x}dx\right)+f\left(x,t\right)dx$$

where *y*(*x,t*) is the transverse displacement and *ρ_l_*(*x*) is the density of the selected segment. The bending moment and the shear force satisfy the following relationships:

(5) $$M=EIy^{\prime \prime }\left(x,t\right)$$

(6) $$F_{S}=\frac{\partial M}{\partial x}=EIy^{\left(3\right)}\left(x,t\right)$$

(7) $$\frac{\partial F_{S}}{\partial x}=\frac{\partial^{2}M}{\partial x^{2}}=EIy^{\left(4\right)}\left(x,t\right)$$

In the free-vibration state, *f*(*x,t*) = 0. We substitute Equations (5)–(7) into Equation (4), and separate time parameter and space parameter (i.e., *y*(*x,t*) = *Y*(*x*)*F*(*t*)), and obtain:

(8) $$-\frac{1}{F\left(t\right)}\frac{d^{2}F\left(t\right)}{dt^{2}}=\frac{1}{\rho_{l}\left(x\right)Y\left(x\right)}\frac{d^{2}}{dx^{2}}\left[EI\frac{d^{2}Y\left(x\right)}{dx^{2}}\right]=\omega^{2}$$

In the above equation, *ρ_l_*(*x*) = *ρS*(*x*) = *ρbdx*. We also substitute Equation (3) into Equation (8). Therefore, it can be concluded that the thickness of the vibrator *b* is positively correlated to the intrinsic frequency ω of the cantilever beam, and show the same conclusion as a previous article [38]. Since it is an inverted cantilever beam bending model, the influence of the inertia moment of the cantilever beam on the natural frequency is comprehensively considered. At the same time, the influence of the boundary constraints on the cantilever beam is also considered comprehensively. Therefore, we conduct the following analysis.

Under the transverse vibration condition of *f* = 3.0 Hz and *A* = 50 mm, the cantilever beam is forced to vibrate under an external load. The voltage output of CB-TENG is shown in Figure 3c. As can be seen, the output voltage of CB-TENG increases with the thickness of the cantilever beam when the thickness is less than 0.12 mm. However, when the thickness of the spring steel exceeds 0.12 mm, the electrical output of CB-TENG shows a decreasing trend. The reason for this phenomenon is that the forced vibration of the cantilever beam is closely related to its thickness and natural frequency. In the thickness range of 0.09 mm to 0.12 mm, the natural frequency of the vibrator increases with the increase in the thickness of the cantilever beam, and gradually approaches the frequency of the external load. As a result, the vibration amplitude of the vibrator, as well as the contact area between the PTFE and the Al electrode, is enlarged when the thickness of the cantilever beam increases. As the thickness of the vibrator is further increased, its stiffness increases and the bending deformation decreases. Therefore, its contact area with the metal plate decreases and the open-circuit voltage presents a downward trend. Overall, the output performance of CB-TENG shows a trend of first increasing and then decreasing with the increase in the thickness of the vibrator, and the electrical output is optimal when the thickness of the spring steel is set to 0.12mm. At this time, the output open-circuit voltage, short-circuit current, and transferred charge reach 245 V, 10.1 μA, and 135 nC, respectively. Therefore, the spring steel with a thickness of 0.12 mm is adopted as the cantilever beam of CB-TENG.

**Figure 4:**
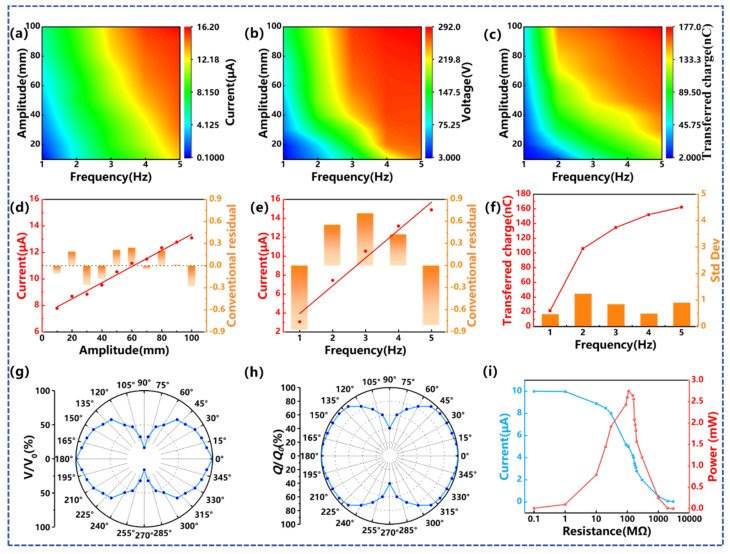
The performance of CB-TENG under different vibration parameters. (**a**) 3D contour of short-circuit current variation with the vibration amplitude and frequency; (**b**) 3D contour of open-circuit voltage variation with the vibration amplitude and frequency; (**c**) 3D contour of transferred charge variation with the vibration amplitude and frequency; (**d**) the short-circuit current variation with the vibration amplitude; (**e**) the short-circuit current variation with the vibration frequency; (**f**) the transferred charge variation with the vibration amplitude; the percentage of (**g**) open-circuit voltage and (**h**) transferred charge varying with azimuth; (**i**) dependence of the voltage and output power density on the external load resistance for the CB-TENG working at *f* = 3 Hz, *A* = 50 mm.

Next, the output performance of CB-TENG under different vibration conditions is tested. The vibration frequency and amplitude range from 1.0 Hz to 5.0 Hz and 10 mm to 100 mm, respectively. The results are illustrated in Figure 4a–c. Apparently, the open-circuit voltage, short-circuit current, and transferred charge of CB-TENG are enhanced with the increase in vibration amplitude and frequency. Under the maximum vibration condition, i.e., *f* = 5.0 Hz and *A* = 100 mm, the specific electrical output parameters are *V_max_* = 292 V, *I_max_* = 16 μA, and *Q_max_* = 177 nC. In particular, the short-circuit current of CB-TENG follows a positive linear relationship with both the vibration frequency and amplitude. As depicted in Figure 4d,e, the correlation coefficients after linear fitting are 0.964 and 0.985, respectively. At a fixed frequency of 3.0 Hz, the short-circuit current increases from 7.9 μA to 12.6 μA as the vibration amplitude increases from 10 mm to 100 mm, while at a fixed amplitude of 50 mm, the short-circuit current increases from 3.1 μA to 15.4 μA with an increasing vibration frequency from 1.0 Hz to 5.0 Hz. Figure 4f shows the relationship between the transferred charge and the vibration frequency at a fixed vibration amplitude of 50 mm. When the vibration frequency increases from 1.0 Hz to 2.0 Hz, the amount of transferred charge increases rapidly from 20 nC to 110 nC. As the vibration frequency continues to increase, the growth rate of the transferred charge decreases significantly. This is due to the fact that at a non-resonance frequency, there is low-amplitude vibration and thus the charge flow is smaller, whereas close to resonance, the charge flow is higher due to higher amplitude vibration and contact initiation.

Affected by the geological environment of drilling, the azimuth angle of the transverse vibration of the drill pipe changes continuously. To verify the output performance of CB-TENG at different azimuth angles, an experimental setup as shown in Figure S3 is established. Considering the symmetry of the circumference, only the electrical output at the azimuth angles of 0° to 90° is tested. Notably, to highlight the effect of the vibration azimuth, the measurement value at 0° is defined as the reference value and is denoted by the subscript 0. As shown in Figure 4g,h, the ordinates represent the ratios of open-circuit voltage and transferred charge to the reference values, respectively. It turns out that the azimuth angle has a significant influence on the output performance of CB-TENG. Both the open-circuit voltage and the transferred charge continue decreasing with the increase in the vibration azimuth. Specifically, the *V_oc_* decreases to 49.9% of the reference voltage at the azimuth angle of 70°, and when the CB-TENG is perpendicular to the vibration direction, the *V_oc_* only accounts for 16.22% of the reference value. For the transferred charge, the attenuation does not exceed 6% until the azimuth angle reaches 50°, and the largest drop occurs with the azimuth angle of 90°, where the amount of transferred charge is 40.5% of the reference value. Figure 4i shows the peak power of CB-TENG by applying different external load resistances. When the external load resistance is 160 MΩ, the peak power can reach 2.56 mW.

**Figure 5:**
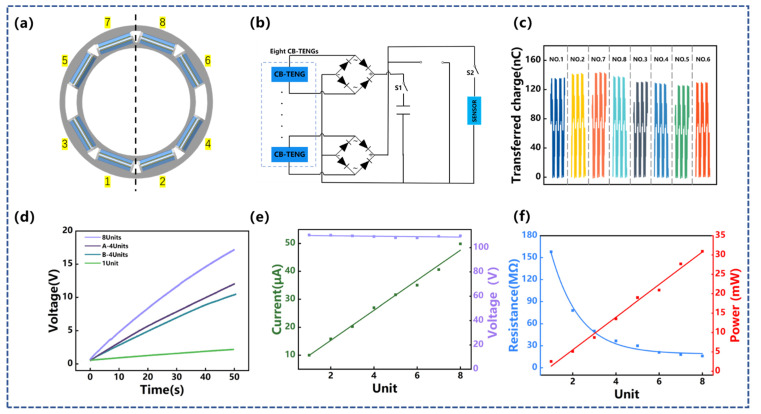
The output performance of the array-type CB-TENG for vibration energy harvesting: (**a**) Array-type CB-TENG layout diagram; (**b**) the working circuit of array-type CB-TENG for vibration energy harvesting to power sensor or testing; (**c**) transferred charge for each TENG unit in the CB- array-type TENG; (**d**) charging performances to a capacitor of 10 μF for different CB-TENG arrays; (**e**) open-circuit voltage and short-circuit current with different amounts of integrated units; (**f**) the output power and the external load resistance with different amounts of integrated units working at *f* = 3 Hz, *A* = 50 mm.

To further utilize the internal space of the drill pipe and improve the electrical output, eight CB-TENGs are integrated into the drill pipe along the circumference to form an array-type vibration-harvesting device, as displayed in Figure 5a. According to the vibration azimuth, the eight CB-TENGs are divided into two groups, in which the CB-TENGs of No.1, No.2, No.7, and No.8 are classified as Group A and the CB-TENGs of No.3, No.4, No.5, and No.6 are classified as Group B. Since the electrical phase for each CB-TENG is different, a rectifier bridge is used to convert the alternating current into direct current and then connect them in parallel (Figure 5b). The results in Figure 5c further confirm the influence of the vibration azimuth and show that the average transferred charges of Group A and Group B are 137.65 nC and 126.3 nC, respectively. For different CB-TENG arrays, the rectified total transferred charges increase linearly with time, as shown in Figure 5d. This is because the transferred charge of array-type CB-TENG increases as the number of arrays increases [39]. The relationship between the short-circuit current and open-circuit voltage with the number of CB-TENG is exhibited in Figure 4e. According to Kirchhoff’s voltage law, the voltage of the power supply is equal to the voltage of each unit. The open-circuit voltage hardly changes with the increase in the number of CB-TENG, which is favorable for parallel electrical connections. According to Kirchhoff’s current law, the current of the power supply is equal to the algebraic sum of the current of each unit. The short-circuit current yields a fairly linear trend with the number of CB-TENGs (*R^2^* = 0.988). More specifically, as the number of CB-TENGs increases from one to eight, the short-circuit current increases from 10 μA to 49.85 μA. In addition, the influence of the number of CB-TENGs on the optimal load resistance and the maximum peak power is further studied, and the results are shown in Figure 5e. The maximum peak power increases linearly from 2.528 mW to 30.95 mW with *R^2^* = 0.962. However, the optimal matching load resistance decreases rapidly as the number of CB-TENGs increases. This is because the CB-TENGs are connected in parallel and the load resistance of each CB-TENG is constant, so the matching resistance of the array-type device decreases exponentially with the number of integrated units [39].

### 3.2. Demonstration Application

**Figure 6:**
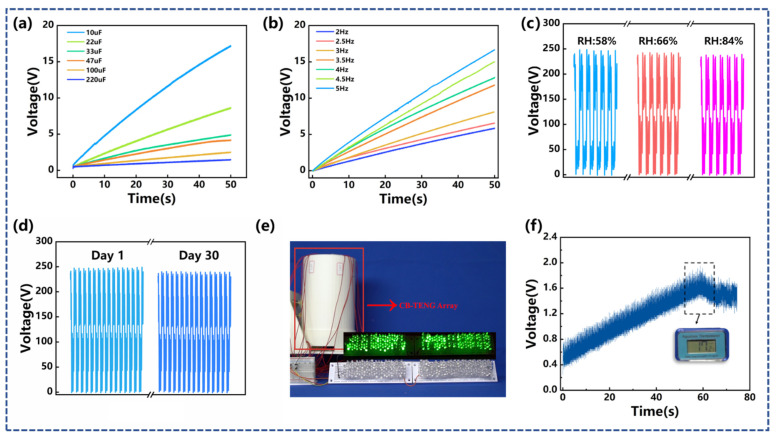
Demonstration applications: (**a**) Voltage of different capacitors (C = 10, 22, 33, 47, 100, and 220 µF) charged by array-type CB-TENG at *f* = 3 Hz, *A* = 50 mm; (**b**) voltage of the same capacitor (C = 33 µF) charged by array-type CB-TENG at different vibration frequency and the same vibration amplitude of 50 mm; (**c**) sensitivity of CB-TENG to relative humidity; (**d**) durability of the CB-TENG; (**e**) the array-type CB-TENG lighting 204 LEDs; (**f**) powering a temperature sensor with array-type CB-TENG.

A series of 50 s charging experiments were performed using the management circuit displayed in Figure 5b. As shown in Figure 6a, in the vibration condition of *f* = 3.0 Hz and *A* = 50 mm, the capacitors with the capacitance of 10 µF, 22 µF, 33 µF, 47 µF, 100 µF, and 200 µF reached 17 V, 8.58 V, 4.92 V, 4.22 V, 2.52 V, and 1.47 V, respectively. Figure 6b shows the effect of vibration frequency on the charging performance. The vibration amplitude of the external load was maintained at 50 mm. When the vibration frequency was 2.0 Hz, 2.5 Hz, 3.0 Hz, 3.5 Hz, 4.0 Hz, 4.5 Hz, and 5.0 Hz, the array-type vibration energy-harvesting device charged the capacitor with a capacitance of 33 µF to 5.86 V, 6.53 V, 8.61V, 12.1 V, 13.41 V, 15.74 V, and 17.16 V, respectively, within 50 s. In addition, humidity sensitivity verification was carried out. It can be seen from Figure 6c that, when the relative humidity of the environment increases from 58% to 65% and 84%, the open-circuit voltage of CB-TENG only yields a reduction of approximately 2% and 5%. The durability test result of CB-TENG is shown in Figure 6d. Throughout the 30-day test, the electrical output decreased by 2%, which confirms the robustness of the device. Figure 6c demonstrates that 204 LED lights are successfully lit by the array-type triboelectric nanogenerator device at the vibration condition of *f* = 3 Hz and *A* = 50 mm. The experimental results can also be found in the Supplementary Video S2. Furthermore, it can be seen from Figure 6f and the Supplementary Video S3 that the array-type triboelectric nanogenerator device can power a common temperature sensor after charging a 100 µF capacitor for 60 s. Therefore, it can be proved that the as-proposed CB-TENG device has certain applicability.

## 4. Conclusions

This study is devoted to developing a cantilever-beam-based triboelectric nanogenerator (CB-TENG) for harvesting transverse vibration energy of the drill pipe in the process of subsea oil and gas exploitation. The vibrator structure formed by the cantilever beam in CB-TENG was characterized by its high aspect ratio and realized effective utilization of the internal space of the drill pipe. Mechanical theoretical analysis and experimental research were conducted to optimize the structure of CB-TENG concerning the backplane structure and the number and thickness of the vibrator. The maximum peak power of CB-TENG can reach 2.56 mW under the typical vibration condition (i.e., *f* = 3.0 Hz and *A* = 50 mm). Within the vibration frequency of 0~5.0 Hz and the amplitude of 0~100 mm, the electrical output of CB-TENG was enhanced as the vibration parameters increased. The open-circuit voltage, short-circuit current, and transferred charge were *V_max_* = 292 V, *I_max_* = 16 μA, and *Q_max_* = 177 nC, respectively, in the maximum vibration condition. Aiming to fully adapt to the internal structure of the drill pipe and further improve the electrical output, eight CB-TENGs were integrated along the circumference to form an array-type vibration energy-harvesting device. Under the vibration condition of *f* = 3.0 Hz and *A* = 50 mm, the short-circuit current and the peak power of the device were 49.85 μA and 30.95 mW, respectively, and the feasibility was demonstrated in terms of charging capacitors, lighting LEDs, and powering sensors. Furthermore, the environmental humidity and robustness tests confirmed that the array-type triboelectric nanogenerator device has certain applicability. The CB-TENG is expected to be a new method of downhole power supply or a supplement to the existing one.

## Figures and Tables

**Table 1:** The conditions and performance of different TENG-based vibration harvesters.

Ref.	Frequency Range/Hz	Amplitude Range/mm	Maximum Voltage/V	Maximum Current/μA	Maximum Charges/nC	Maximum Power/μW	Maximum Power Density	Response Frequency/Hz
[31]	4–10	4	102	-	-	14	5.56 mW/m^2^	8
[29]	VE ^1^: 5–30 HE ^2^: 2.5–30	1–20	83	5	30	-	240 mW/m^2^ 45mW/m^2^	VE: 16 HE: 8.5
[32]	10–60	1–4.5	98	3.4	28.5	-	50 W/m^3^	25
[13]	0.4–2.0	50–130	-	43.53	3890	-	49 W/m^3^	2
[30]	0–5	-	10	55.7	-	-	252.3 mW/m^3^	3.7
[35]	2.5	4–8	428	114	-	-	4.32 W/m^2^	2.5
This work	0–5	10–100	113	49.85	-	30950	-	3

^1^ Vertical Excitation. ^2^ Horizontal Excitation.

## Data Availability

The data presented in this study are available on request from the corresponding author.

## Supplementary Materials

The following supporting information can be downloaded at: https://www.mdpi.com/article/10.3390/s22114287/s1, Figure S1: Simulations of electric potential distributions for the CB-TENG; Figure S2: The output of CB-TENG with different thicknesses of spring steel; Figure S3: The experimental setup of azimuth angle test. Video S1: The specific vibration mode of CB-TENG; Video S2: 204 LEDs are lit up by array-type CB-TENG; Video S3: The array-type CB-TENG powers a sensor.

## References

[1] Tong X., Zhang G., Wang Z., Wen Z., Tian Z., Wang H., Ma F., Wu Y. (2018). Distribution and potential of global oil and gas resources. Pet. Explor. Dev..

[2] Nakken E.I., Mjaaland S., Solstad A. A New MWD Concept for Geological Positioning of Horizontal Wells. Proceedings of the SPE Annual Technical Conference and Exhibition.

[3] Fripp M.L., Hamid S., Moore D.W., Kyle D., Caja J., Dunstan T.D. Development of a High-Temperature Rechargeable Battery for Downhole Use in Petroleum Industry. Proceedings of the Offshore Technology Conference.

[4] Wang Z., Jian Z., Guo Y., Zhu W., Wang H. The Turbine Parameter Study of Down-hole Turbine Generator of While Drilling for Exploring of China Sea. Proceedings of the Twenty-Fourth International Ocean and Polar Engineering Conference.

[5] Gooneratne C.P., Li B., Deffenbaugh M., Moellendick T. (2019). Downhole Communication and Power Supplies to Instruments and Communication Modules. Instruments, Measurement Principles and Communication Technologies for Downhole Drilling Environments.

[6] Chen J., Wang Y. (2019). A dual electromagnetic array with intrinsic frequency up-conversion for broadband vibrational energy harvesting. Appl. Phys. Lett..

[7] Naifar S., Bradai S., Viehweger C., Kanoun O. (2017). Survey of electromagnetic and magnetoelectric vibration energy harvesters for low frequency excitation. Measurement.

[8] Remick K., Dane Quinn D., Michael McFarland D., Bergman L., Vakakis A. (2016). High-frequency vibration energy harvesting from impulsive excitation utilizing intentional dynamic instability caused by strong nonlinearity. J. Sound Vib..

[9] Saha C.R., Donnell T.O., Loder H., Beeby S., Tudor J. (2006). Optimization of an Electromagnetic Energy Harvesting Device. IEEE Trans. Magn..

[10] Swallow L.M., Luo J.K., Siores E., Patel I., Dodds D. (2008). A piezoelectric fibre composite based energy harvesting device for potential wearable applications. Smart Mater. Struct..

[11] Peigney M., Siegert D. (2013). Piezoelectric energy harvesting from traffic-induced bridge vibrations. Smart Mater. Struct..

[12] Delnavaz A., Voix J. (2014). Flexible piezoelectric energy harvesting from jaw movements. Smart Mater. Struct..

[13] Wang H., Zhu C., Wang W., Xu R., Chen P., Du T., Xue T., Wang Z., Xu M. (2022). A Stackable Triboelectric Nanogenerator for Wave-Driven Marine Buoys. Nanomaterials.

[14] Chang J., Zhu C., Wang Z., Wang Y., Li C., Hu Q., Xu R., Du T., Xu M., Feng L. (2022). A full-set and self-powered ammonia leakage monitor system based on CNTs-PPy and triboelectric nanogenerator for zero-carbon vessels. Nano Energy.

[15] Liu L., Guo X., Lee C. (2021). Promoting smart cities into the 5G era with multi-field Internet of Things (IoT) applications powered with advanced mechanical energy harvesters. Nano Energy.

[16] Carneiro P., Soares dos Santos M.P., Rodrigues A., Ferreira J.A.F., Simões J.A.O., Marques A.T., Kholkin A.L. (2020). Electromagnetic energy harvesting using magnetic levitation architectures: A review. Appl. Energy.

[17] Dong L., Closson A.B., Jin C., Trase I., Chen Z., Zhang J.X.J. (2019). Vibration-Energy-Harvesting System: Transduction Mechanisms, Frequency Tuning Techniques, and Biomechanical Applications. Adv. Mater. Technol..

[18] Lei H., Cao K., Chen Y., Liang Z., Wen Z., Jiang L., Sun X. (2022). 3D-printed endoplasmic reticulum rGO microstructure based self-powered triboelectric pressure sensor. Chem. Eng. J..

[19] Lei H., Xiao J., Chen Y., Jiang J., Xu R., Wen Z., Dong B., Sun X. (2022). Bamboo-inspired self-powered triboelectric sensor for touch sensing and sitting posture monitoring. Nano Energy.

[20] Jiang T., Zhang L.M., Chen X., Han C.B., Tang W., Zhang C., Xu L., Wang Z.L. (2015). Structural Optimization of Triboelectric Nanogenerator for Harvesting Water Wave Energy. ACS Nano.

[21] Liang X., Jiang T., Liu G., Xiao T., Xu L., Li W., Xi F., Zhang C., Wang Z.L. (2019). Triboelectric Nanogenerator Networks Integrated with Power Management Module for Water Wave Energy Harvesting. Adv. Funct. Mater..

[22] Liang X., Jiang T., Liu G., Feng Y., Zhang C., Wang Z.L. (2020). Spherical triboelectric nanogenerator integrated with power management module for harvesting multidirectional water wave energy. Energy Environ. Sci..

[23] Zhang L.M., Han C.B., Jiang T., Zhou T., Li X.H., Zhang C., Wang Z.L. (2016). Multilayer wavy-structured robust triboelectric nanogenerator for harvesting water wave energy. Nano Energy.

[24] Liu J., Wen Z., Lei H., Gao Z., Sun X. (2022). A Liquid–Solid Interface-Based Triboelectric Tactile Sensor with Ultrahigh Sensitivity of 21.48 kPa^−1^. Nano-Micro Lett..

[25] Chen C., Wen Z., Shi J., Jian X., Li P., Yeow J.T.W., Sun X. (2020). Micro triboelectric ultrasonic device for acoustic energy transfer and signal communication. Nat. Commun..

[26] Ma M., Kang Z., Liao Q., Zhang Q., Gao F., Zhao X., Zhang Z., Zhang Y. (2018). Development, applications, and future directions of triboelectric nanogenerators. Nano Res..

[27] Qi Y., Liu G., Gao Y., Bu T., Zhang X., Xu C., Lin Y., Zhang C. (2021). Frequency Band Characteristics of a Triboelectric Nanogenerator and Ultra-Wide-Band Vibrational Energy Harvesting. ACS Appl. Mater. Interfaces.

[28] Chen J., Zhu G., Yang W., Jing Q., Bai P., Yang Y., Hou T.-C., Wang Z.L. (2013). Harmonic-Resonator-Based Triboelectric Nanogenerator as a Sustainable Power Source and a Self-Powered Active Vibration Sensor. Adv. Mater..

[29] Xu M., Wang P., Wang Y.-C., Zhang S.L., Wang A.C., Zhang C., Wang Z., Pan X., Wang Z.L. (2018). A Soft and Robust Spring Based Triboelectric Nanogenerator for Harvesting Arbitrary Directional Vibration Energy and Self-Powered Vibration Sensing. Adv. Energy Mater..

[30] Yang W., Chen J., Zhu G., Wen X., Bai P., Su Y., Lin Y., Wang Z. (2013). Harvesting vibration energy by a triple-cantilever based triboelectric nanogenerator. Nano Res..

[31] Li R., Zhang H., Wang L., Liu G. (2021). A Contact-Mode Triboelectric Nanogenerator for Energy Harvesting from Marine Pipe Vibrations. Sensors.

[32] Xiao X., Zhang X., Wang S., Ouyang H., Chen P., Song L., Yuan H., Ji Y., Wang P., Li Z. (2019). Honeycomb Structure Inspired Triboelectric Nanogenerator for Highly Effective Vibration Energy Harvesting and Self-Powered Engine Condition Monitoring. Adv. Energy Mater..

[33] Du T., Zuo X., Dong F., Li S., Mtui A.E., Zou Y., Zhang P., Zhao J., Zhang Y., Sun P. (2021). A Self-Powered and Highly Accurate Vibration Sensor Based on Bouncing-Ball Triboelectric Nanogenerator for Intelligent Ship Machinery Monitoring. Micromachines.

[34] Wu C., Huang H., Yang S., Wen G. (2020). Pagoda-Shaped Triboelectric Nanogenerator With High Reliability for Harvesting Vibration Energy and Measuring Vibration Frequency in Downhole. IEEE Sens. J..

[35] Liu G., Xu W., Xia X., Shi H., Hu C. (2015). Newton’s cradle motion-like triboelectric nanogenerator to enhance energy recycle efficiency by utilizing elastic deformation. J. Mater. Chem. A.

[36] Toprak A., Tigli O. (2014). Piezoelectric energy harvesting: State-of-the-art and challenges. Appl. Phys. Rev..

[37] Zou Y., Xu J., Chen K., Chen J. (2021). Advances in Nanostructures for High-Performance Triboelectric Nanogenerators. Adv. Mater. Technol..

[38] Kim W., Bhatia D., Jeong S., Choi D. (2019). Mechanical energy conversion systems for triboelectric nanogenerators: Kinematic and vibrational designs. Nano Energy.

[39] Liu W., Xu L., Bu T., Yang H., Liu G., Li W., Pang Y., Hu C., Zhang C., Cheng T. (2019). Torus structured triboelectric nanogenerator array for water wave energy harvesting. Nano Energy.

