# Supplementary material for: A Cantilever Beam-Based Triboelectric Nanogenerator as a Drill Pipe Transverse Vibration Energy Harvester Powering Intelligent Exploitation System
Source: Sensors (Basel). 2022 Jun 4;22(11):4287. doi: 10.3390/s22114287 (PMC9185564; doi:10.3390/s22114287)
Supplement: Supplementary file 1 [file sensors-22-04287-s001.zip › sensors-1745033-SI.pdf]

sensors

MDPI

Supplementary Materials

A Cantilever Beam-Based Triboelectric Nanogenerator as a Drill Pipe Transverse Vibration Energy Harvester Powering Intelligent Exploitation System

Zhenhui Lian, Qunyi Wang, Chuanqing Zhu, Cong Zhao, Qiang Zhao, Yan Wang, Zhiyuan Hu, Ruijiang Xu, Yukai Lin, Tianyu Chen, Xiangyu Liu, Xiaoyan Xu, Ling Liu, Xiu Xiao * and Minyi Xu

Dalian Key Lab of Marine Micro/Nano Energy and Self-Powered Systems, Marine Engineering College, Dalian Maritime University, Dalian 116026, China; zhlian_bkpp@dlmu.edu.cn (Z.L.); 1120211168@dlmu.edu.cn (Q.W.); zcq@dlmu.edu.cn (C.Z.); zhaocong@dlmu.edu.cn (C.Z.); zhao1989@bcnu.edu.cn (Q.Z.); wangyanme@dlmu.edu.cn (Y.W.); zhiyuanhu@dlmu.edu.cn (Z.H.); xuruijiang@dlmu.edu.cn (R.X.); linyukai@dlmu.edu.cn (Y.L.); cty@dlmu.edu.cn (T.C.); simonlxy@dlmu.edu.cn (X.L.); yz1769135517@dlmu.edu.cn (X.X.); pinky@dlmu.edu.cn (L.L.); xuminyi@dlmu.edu.cn (M.X.).

* Correspondence: xiaoxiu@dlmu.edu.cn; Tel.: +86-13610865112

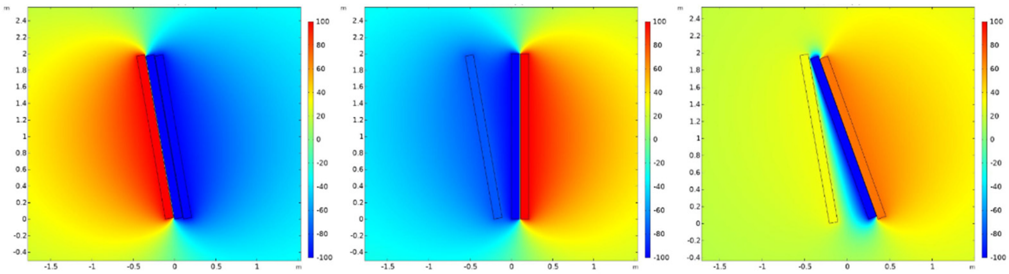

Figure S1. Simulations of electric potential distributions for CB-TENG.

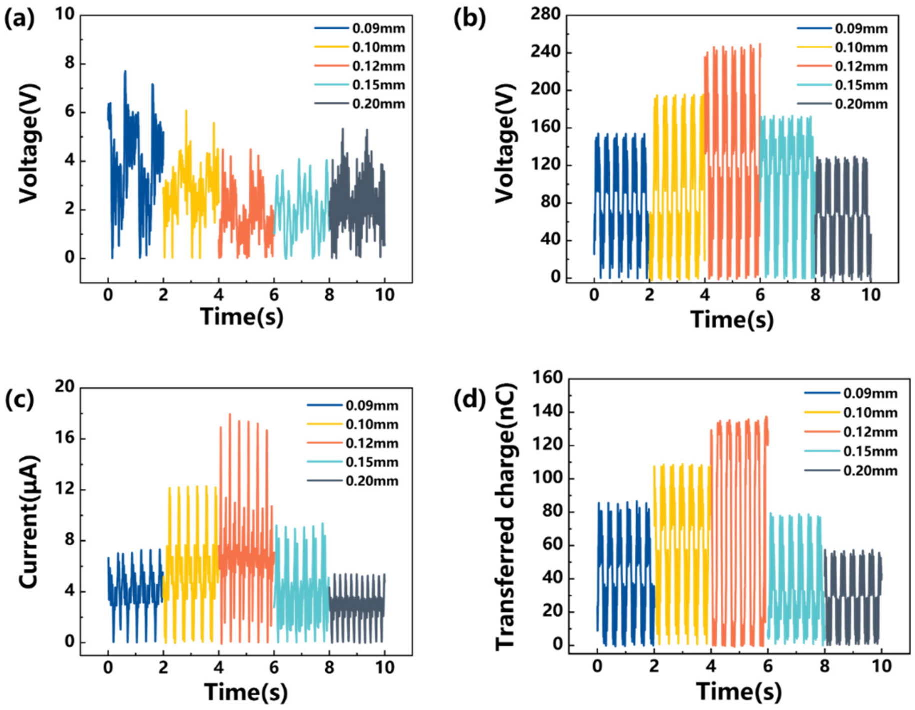

Figure S2. The output of CB-TENG with different thickness of spring steel. (a) The open-circuit voltage of CB-TENG at *f*=1.0 HZ,*A*=10 mm with different thickness of spring steel; (b) The open-circuit voltage of CB-TENG at *f*=3HZ *A*=50mm with different thickness of spring steel; (c) The short-circuit current of CB-TENG at *f*=3HZ *A*=50mm with different thickness of spring steel; (d) The transferred charge of CB-TENG at *f*=3HZ *A*=50mm with different thickness of spring steel.

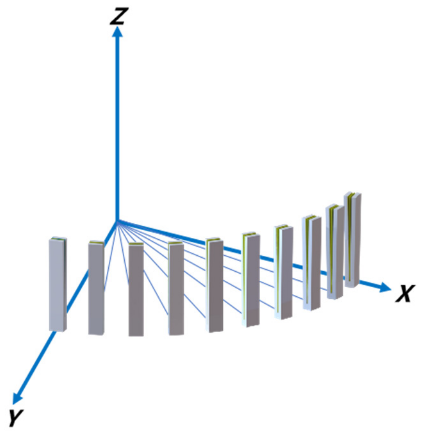

Figure S3. The experimental setup of azimuth angle test.

The following demo videos are also uploaded:

Supplementary Video S1: The specific vibration mode of CB-TENG.

Supplementary Video S2: 204 LEDs are lighted up by array-type CB-TENG.

Supplementary Video S3: The array-type CB-TENG powers a sensor.
